# Long-term Impact of E-cigarette and Vaping Product Use-associated Lung Injury on Diffusing Capacity for Carbon Monoxide Values: A Case Series

**DOI:** 10.7759/cureus.7002

**Published:** 2020-02-15

**Authors:** Mudassar Ahmad, Ghulam Aftab, Sana Rehman, Douglas Frenia

**Affiliations:** 1 Pulmonary Medicine, Saint Peter's University Hospital / Rutgers University, New Brunswick, USA; 2 Internal Medicine, Orange Park Medical Center, Orange Park, USA; 3 Internal Medicine, Shaikh Khalifa Bin Zayed Al-Nahyan Medical and Dental College, Lahore, PAK; 4 Pulmonary Critical Care, Saint Peter’s University Hospital, New Brunswick, USA

**Keywords:** e-cigarette and vaping product use associated lung injury (evali), pulmonary function testing

## Abstract

There has been an outbreak of lung injury associated with e-cigarettes and vaping in the United States since early 2019. We present two cases who were admitted to the hospital with shortness of breath and cough. Chest imaging showed they had interstitial changes. They were diagnosed with e-cigarette and vaping product use-associated lung injury (EVALI) and treated with steroids and supportive management. With an improvement in symptoms, they were discharged home.

On follow-up in the clinic, both patients were asymptomatic and had complete resolution of radiographic abnormalities. However, pulmonary function testing showed reduced diffusion capacity for carbon monoxide (DLCO). Total lung capacity (TLC), forced vital capacity (FVC), forced expiratory volume in the first one second (FEV-1), and the FEV-1/FVC ratio were normal.

## Introduction

There has been an outbreak of lung injury associated with e-cigarettes and vaping in the United States since early 2019. As of January 21, 2020, the Centers for Disease Control (CDC) has received reports of 2,711 cases of e-cigarette and vaping product use-associated lung injury (EVALI) [1]. The exact cause of lung injury remains unclear, but patterns are consistent with a toxic inhalation pulmonary injury, which suggests a direct injury rather than an infectious cause [2]. Vitamin E acetate is identified as a chemical of concern by the CDC among people with EVALI. This agent is used for thickening in tetrahydrocannabinol (THC)-containing e-cigarette and vaping products [1]. Since the outbreak of EVALI, most research has focused on the diagnosis and acute management of EVALI [3-4]. The long-lasting effects of EVALI have yet to be thoroughly investigated. We present two cases that were discharged from the hospital after recovering from EVALI but had reduced diffusion capacity for carbon monoxide (DLCO) in follow-up pulmonary function testing.

## Case presentation

Case 1

**Figure 1:**
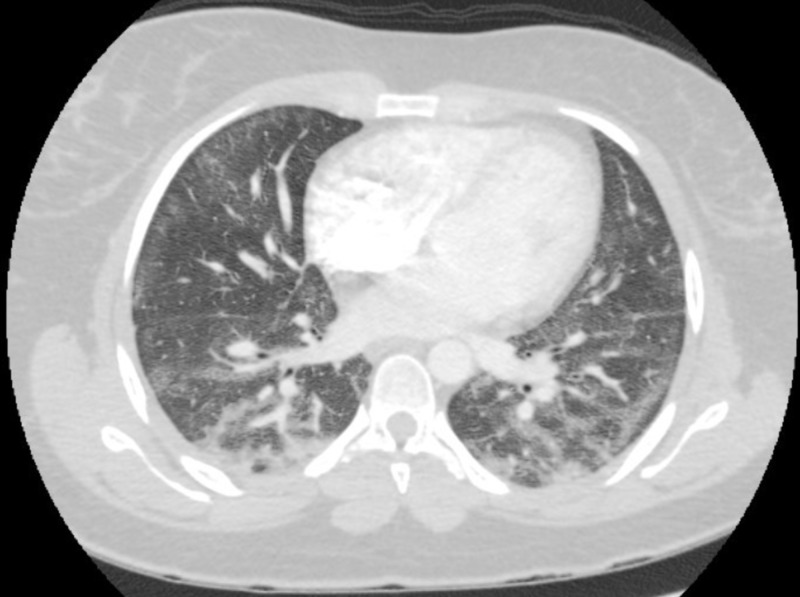
CTA chest showing bilateral ground-glass opacities CTA: Computed tomography angiography

**Figure 2:**
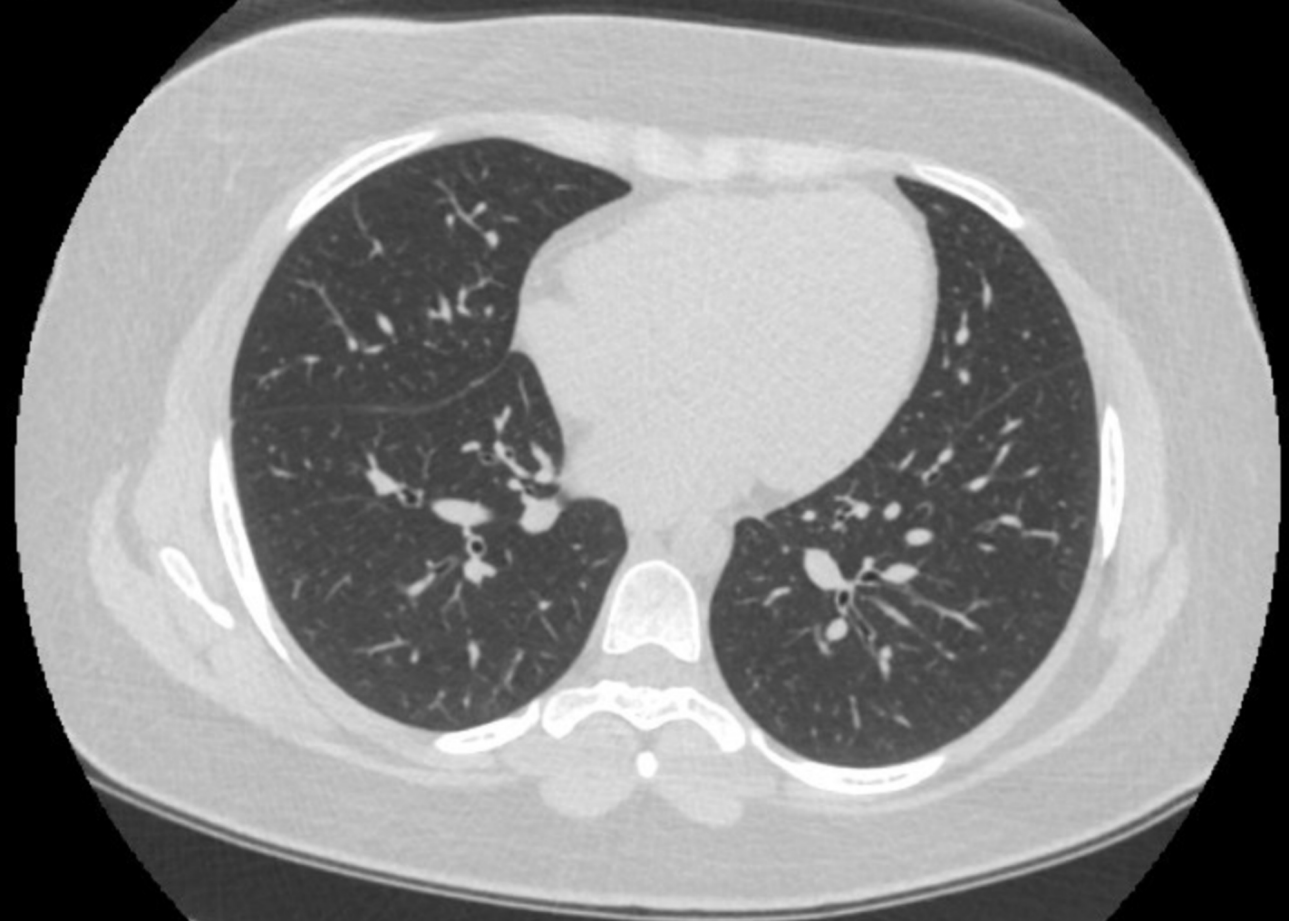
Follow-up CT chest after two months CT: Computed tomography

A 23-year-old female presented with the chief complaint of shortness of breath, dry cough, and low-grade fevers. She reported a history of smoking cigarettes and marijuana and had started vaping recently. She denied any rash, hemoptysis, joint pains, recent travel, intravenous drug use, sick contacts, or previous history of tuberculosis. The patient had initial workup in the emergency department (ED), including chest imaging and basic lab testing. A computed tomography angiography (CTA) of the chest showed bilateral ground-glass opacifications, which were more pronounced in the lower lobes, along with mediastinal and hilar lymphadenopathy (Figure 1). Laboratory findings were significant for an elevated white blood cell (WBC) count without bandemia. Blood cultures, respiratory viral panel, influenza testing, Legionella, and strep urine antigens were negative. She was empirically started on treatment for community-acquired pneumonia with ceftriaxone and azithromycin. Despite being on antibiotics, she continued to spike fevers and had a few episodes of vomiting during her hospitalization. She was provided supportive treatment, started recovering later, and was discharged to complete a one-week course of antibiotics. The patient met the criteria for a “confirmed case” as per the CDC case definition guidelines [5]. She had follow-up pulmonary function tests (PFTs) performed two months after discharge, which showed a diffusion capacity of 63% of predicted. TLC, FVC, FEV-1, and the FEV-1/FVC ratio were normal. Follow-up computed tomography (CT) after two months showed the resolution of ground-glass opacities (Figure 2).

Case 2

**Figure 3:**
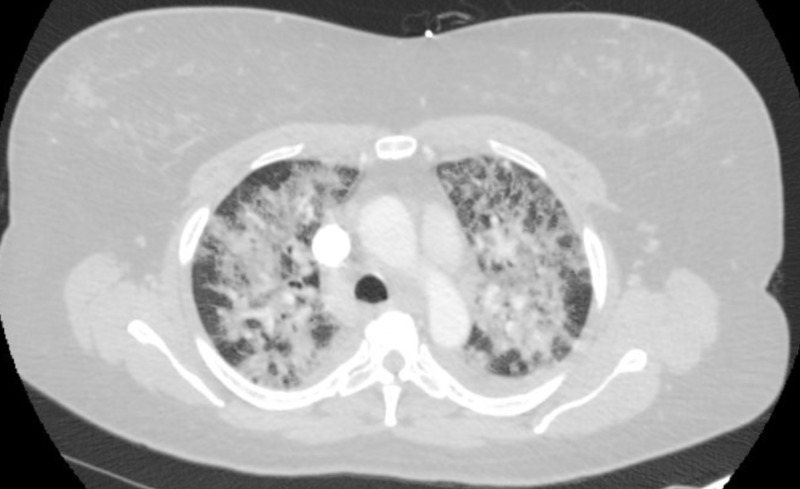
CTA chest showing bilateral alveolar opacities CTA: Computed tomography angiography

A 46-year-old female presented with shortness of breath and associated dry cough for two days. She denied recent travel, sick contacts, fever, chills, night sweats, chest pain, and sputum production, as well as a prior history of lung disease. She stated that she had never smoked or used vaping products. A CTA of the chest was performed, which showed diffuse patchy alveolar opacities throughout both lungs (Figure 3). She was initially placed on a high-flow nasal cannula and broad-spectrum antibiotics, but her condition worsened quickly and she had to be intubated and temporarily paralyzed to help with oxygenation. She was started on high-dose steroids due to concern for acute interstitial lung disease. Upon arrival, the patient had an elevated WBC count with bandemia, as well as an elevated lactic acid of 2.3 mmol/L. She tested negative for human immunodeficiency viruses (HIV). Blood cultures, respiratory viral panel, and influenza testing were negative. Urine Legionella and Streptococcus antigen were also negative. Fiberoptic bronchoscopy with bronchoalveolar lavage (BAL) was performed. The BAL analysis showed the patient had 91% neutrophils. Cultures from the BAL fluid were negative. No cysts of pneumocystis were identified. Oil Red O stain was performed, and it showed positive staining in a small number of alveolar macrophages (<5% of the cellular population present). A basic rheumatologic workup showed an antinuclear antibodies titer of 1:40. Tests for rheumatoid factor and antinuclear cytoplasmic antibodies were negative. An echocardiogram showed normal ejection fraction and there was no valvular abnormality.

**Figure 4:**
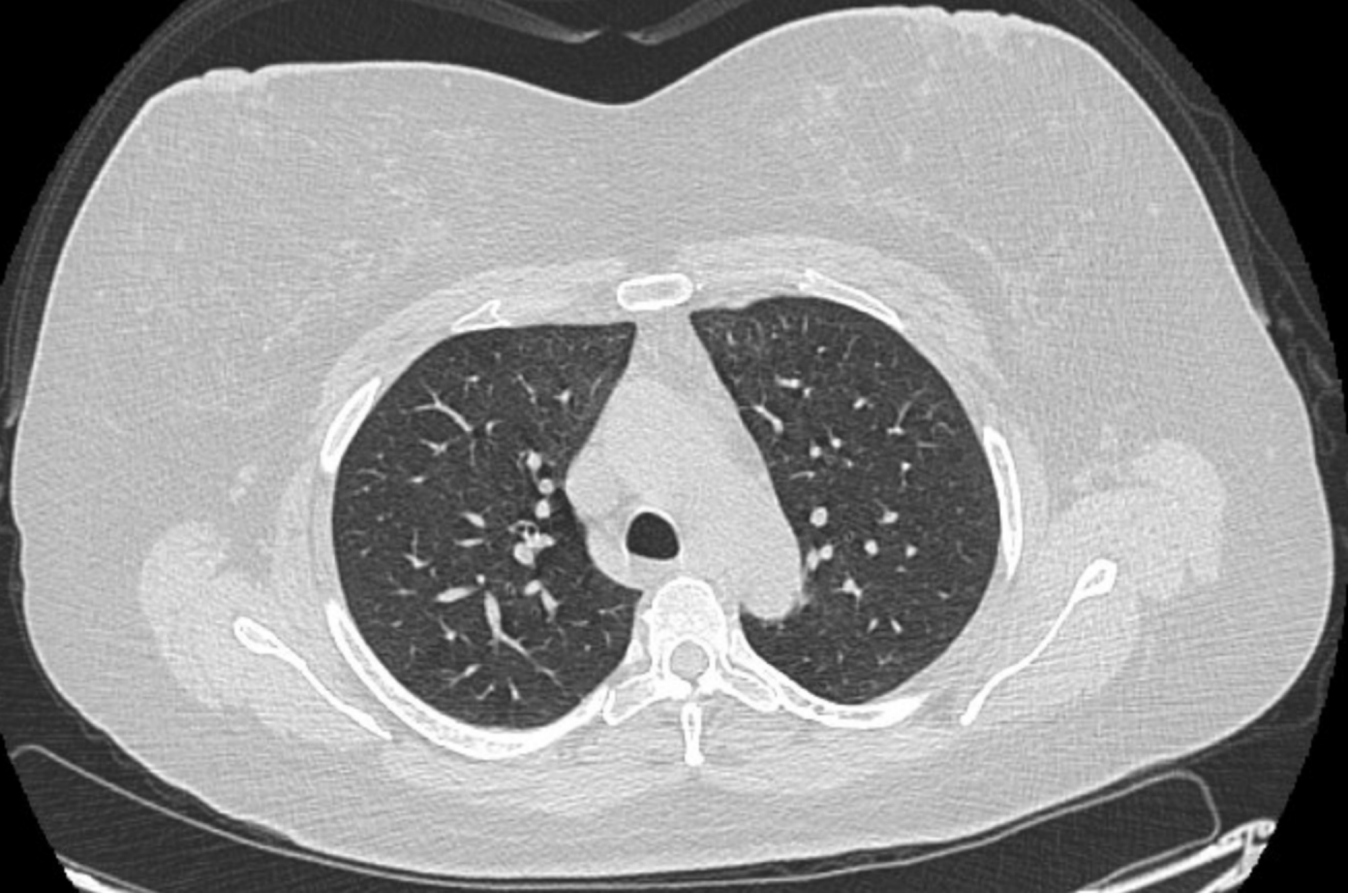
Follow-up CT chest after two months CT: Computed tomography

A few days after intubation, the patient’s mother revealed that the patient, contrary to what she admitted to the hospital staff earlier, had, in fact, been using e-cigarettes one month prior to her hospital admission. Meanwhile, the patient’s condition improved until she was extubated to a nasal cannula after being on the ventilator for five days. She was later transitioned to room air and discharged to a rehabilitation center. She was advised to complete a 10-day-long course of steroids. She also met the criteria for a “confirmed case” of EVALI as per CDC case definition guidelines [3]. She had follow-up PFTs performed, which showed a diffusion capacity of 61% of predicted. TLC, FVC, FEV-1, and the FEV-1/FVC ratio were normal. This was seen despite the resolution of radiographic abnormalities as seen in Figure 4. This is the follow-up of the case published on November 22, 2019 [6].

## Discussion

In all cases presented above, our patients were provided supportive treatment, which included the administration of steroids. This is in line with the current management of patients with EVALI. On follow-up in the clinic, both patients were asymptomatic.

Even though the diagnosis and management of acute lung injury associated with EVALI have been topics of focused investigation, the long-lasting effects of EVALI have not been studied extensively since it is a relatively recent disease [3]. Clinicians should be aware of the possibility of the chronic effects of EVALI on lung functions, especially low DLCO, as documented in these cases. The literature review revealed only one study showing the effects of EVALI on pulmonary function testing. It showed that five of the six patients with abnormal pulmonary function tests had a low DLCO [2]. It is interesting to note that this may hold true despite the resolution of symptoms and radiographic abnormalities. Decreased DLCO has been shown to be a predictor of all-cause mortality independent of other spirometric volumes [7]. It is, therefore, important that patients with EVALI are followed up closely and have pulmonary function testing performed, even if radiographic abnormalities and symptoms have resolved.

As more cases of EVALI are reported, more is expected to be learned about its long-term physiological effects.

## Conclusions

With the recent outbreak of lung injury associated with e-cigarettes and vaping, physicians should be aware of the acute and long-term impact of vaping on the lungs. The diagnosis and management of acute lung injury associated with EVALI have been topics of investigation. As this is a recent phenomenon, the long-lasting effects of EVALI have not been studied thoroughly. Physicians should be aware of the possibility of reduced DLCO in patients who have recovered from acute illness.
